# Economic Evaluation of On-Site Computed Tomography at Major Events Using Data from the Munich Oktoberfest—A German and U.S. Healthcare Perspective

**DOI:** 10.3390/jcm14072375

**Published:** 2025-03-30

**Authors:** Wilhelm Flatz, Viktoria Bogner-Flatz, Dominik Hinzmann, Bazarian J. Jeffrey, Jens Ricke, Kanz Karl-Georg, Wolfgang G. Kunz, Dirk Mehrens

**Affiliations:** 1 Department of Radiology, LMU University Hospital, LMU Munich, 81377 Munich, Germany; jens.ricke@med.uni-muenchen.de (J.R.); wolfgang.kunz@med.uni-muenchen.de (W.G.K.);; 2 Department of Orthopaedics and Trauma Surgery, Musculoskeletal University Center Munich (MUM), LMU University Hospital, LMU Munich, 81377 Munich, Germany; viktoria.bogner-flatz@aelrd-bayern.de; 3 Emergency Medical Service Authority–Upper Bavaria, Government of Upper Bavaria, 80538 Munich, Germany; 4 Department of Clinical Medicine, Department of Anesthesiology and Intensive Care Munich, TUM School of Medicine and Health, 80333 Munich, Germany; dominik.hinzmann.aelrd@rzvmuenchen.de; 5 Emergency Medical Service Authority City of Munich and Greater Area, 80466 Munich, Germany; 6 Department of Emergency Medicine, University of Rochester School of Medicine and Dentistry, Rochester, NY 14642, USA; jeff_bazarian@urmc.rochester.edu; 7 Department of Trauma Surgery, TUM School of Medicine and Health, 80333 Munich, Germany; karl-georg.kanz@outlook.de

**Keywords:** Oktoberfest, CT, computed tomography, on-site, mass event, mass gathering, cost effectiveness, CEA, mTBI, EMS

## Abstract

**Background/Objectives**: The Munich Oktoberfest is the largest Volksfest in the world, attracting more than 6 million visitors every year to the city of Munich and surroundings, posing challenges to healthcare providers. Since 2022, a CT has been installed on the festival site to decrease patient transport to emergency departments (EDs) and relieve EDs of a significant number of patients. The aim of our studies was to determine the economic impact of on-site CT on the healthcare system, both from a German and a U.S. perspective. **Methods**: A decision model was built using patient data from the Munich Oktoberfest. Two scenarios were investigated where patients with mild traumatic brain injuries were either scanned for intracranial hemorrhage (ICH) with CT on-site or brought directly to an ED for further investigation. Costs for patient transportation and CT scans were derived from German national reimbursement rates as well as U.S. Medicare data. Costs were calculated by diagnosis in the national currency. **Results**: In all scenarios, on-site CT scans were associated with reduced costs per patient (EUR 243 vs. EUR 908 in the German setting and EUR 438 vs. EUR 1635 in the international setting, as well as USD 160 vs. USD 403 in the U.S. setting). For the U.S. scenario, the proportion of ICH in the patient group, as well as the transport distance, had the strongest impact on average costs per patient. **Conclusions**: On-site CT scanning is a cost-reducing as well as clinically beneficial method for triaging patients at the Munich Oktoberfest.

## 1. Introduction

During the 14 to 18 days of Munich’s Oktoberfest, the largest Volksfest in the world [1], the city of Munich is host to more than 6 million visitors from all around the world. This poses significant challenges to the emergency medical systems (EMSs) and emergency departments (EDs) of the hospitals in Munich and its surroundings. During this time period, 30% more patients are brought to the different EDs of the city via ambulance alone, not including patients presenting themselves to the emergency departments of the hospitals on their own [2]. In response to this surge, many hospitals within Munich implement strategic measures to enhance their capacity, including increasing medical personnel and security staff, particularly in emergency departments, to manage the heightened patient volume associated with Oktoberfest-related medical incidents.

Simultaneously, it remains imperative to uphold the provision of high-quality medical care for non-Oktoberfest-related emergencies. Hospitals must balance the increased patient load while ensuring that individuals requiring urgent medical attention for conditions unrelated to the festival continue to receive timely and adequate treatment. This necessitates meticulous resource allocation, enhanced co-ordination between medical facilities, and the reinforcement of emergency response systems to maintain overall healthcare service efficiency during this demanding period.

The neurological assessment of patients presenting with conditions related to Oktoberfest can be particularly challenging due to the high prevalence of alcohol intoxication. Acute alcohol consumption can significantly impair cognitive function, level of consciousness, and co-ordination, making it difficult to accurately evaluate neurological status, detect underlying pathologies, or differentiate intoxication from more serious neurological conditions, such as traumatic brain injury, stroke, or metabolic disturbances.

In addition to alcohol-related impairment, a significant proportion of patients requiring medical attention during Oktoberfest present with traumatic injuries. These injuries often result from falls, altercations, or accidents associated with the high-density crowds, festive activities, and overall environment of the event. Lacerations, contusions, fractures, and head injuries are among the most frequently encountered trauma-related diagnoses. Furthermore, the combination of alcohol consumption and physical exertion can contribute to dehydration, cardiovascular strain, and the exacerbation of pre-existing medical conditions, leading to additional medical emergencies.

Each year, thousands of individuals seek or require medical assistance directly on-site at Oktoberfest [3]. These patients receive evaluation and treatment at the designated medical facility on-site (“Sanitätswache”), which is specifically set up to manage a wide range of medical emergencies directly on the event grounds [4]. This facility provides immediate medical intervention, allowing for triage and stabilization before determining whether further high-level hospital-based care is necessary. It is here at the “Sanitätswache”, the specialized medical station in a secured area on the festival grounds, where a highly trained team, consisting of 450 paramedics and 55 physicians, ensures that a wide range of medical emergencies can be promptly assessed and managed on-site [4].

The presence of such a dedicated medical infrastructure is crucial in ensuring timely and effective medical support for festival attendees while alleviating the burden on city hospitals and emergency medical services. The medical personnel at the “Sanitätswache” are equipped to handle various conditions, ranging from minor injuries and alcohol-related complications to more severe medical emergencies requiring urgent intervention. The presence of experienced healthcare professionals enables rapid triage, stabilization, and, when necessary, the co-ordination of patient transport to nearby hospitals for advanced care.

Many patients suffering from mild traumatic brain injuries (mTBIs) and being intoxicated necessitate computed tomography (CT) scanning of the head to exclude severe head trauma [5,6]. The majority of these patients with mTBIs will be discharged from the ED due to negative findings in the head CT scans [5,6,7].

To reduce the burden of necessary CT scans on emergency departments (EDs), we installed an on-site CT scanner at the Munich Oktoberfest for the first time in 2022. The container-based state-of-the-art CT scanner was installed in the area of the “Behördehof”, a highly secured area located at the western side of the Theresienwiese. The Theresienwiese is the area where the festival is located, the festival grounds being 34.5 acres in size [8].

The “Sanitätswache” is strategically located in the “Behördenhof”, a designated area specifically designed to accommodate emergency response teams during Oktoberfest. This government services courtyard serves as a centralized hub where various emergency response professionals work in close collaboration to ensure the safety and well-being of festival attendees.

Within the “Behördenhof”, multiple emergency and public safety services operate in close proximity, facilitating seamless communication and co-ordinated response efforts. These services include emergency medical services (EMSs), the medical treatment unit, the fire department, and law enforcement personnel from the police department. Their combined presence ensures that a wide range of incidents, from medical emergencies and traumatic injuries to fire hazards and security threats, can be effectively managed in a timely manner.

The “Behördenhof” itself is a reinforced concrete building complex that was purposefully constructed by the City of Munich to serve as a fully equipped operational base for first responders during Oktoberfest. This facility provides the necessary infrastructure to support emergency medical care, security operations, and disaster response planning, thereby enhancing the overall safety and efficiency of emergency interventions throughout the festival.

The CT container was deployed in close vicinity to the “Sanitätswache” in order to provide short distances for the patients being examined and to facilitate close communication possibilities between the radiologic team and the medical teams working on-site.

By providing high-quality CT diagnostics on-site, integrated into a dedicated algorithmic approach for clinical patient selection, transport services to nearby EDs were significantly reduced [7]. Due to the encouraging results of successful patient triage and use of the mobile CT scanner on-site in 2022, we repeated the CT setup at the Oktoberfest in the years 2023 and 2024. After successfully implementing the on-site CT, we aimed to investigate the economic impact on the healthcare system associated with providing mobile CT scans on-site at this large-scale event. The objective of this study is to examine the potential cost reductions for different healthcare systems in Germany and the U.S. Additional challenges associated with this project have been discussed elsewhere [9].

## 2. Methods

Approval of the Institutional Review Board of Ludwig-Maximilians-University was waived under IRB Nr. 20-258 for emergency referral data and under IRB Nr. 22-0885 for all other anonymized patient data.

### 2.1. Study Population

We used data gathered from 2022 and 2023 on patients from the Munich Oktoberfest who had an indication for computed tomography (CT) due to head trauma/mTBI.

Visitors of the Oktoberfest who suffered an adequate trauma to the head (mild traumatic brain injury, mTBI) or face were examined by an emergency physician on-site, following a standardized clinical protocol as a decision support tool, which was adapted from a well-established clinical diagnostic algorithm combining NEXUS and Canadian head rule in terms of standard of care [10]. In case of severe head injury determined by GCS or severe trauma mechanisms, patients were immediately transferred to a suitable emergency center, without qualifying for a CT examination on-site.

In total, 498 patients were included. Of these patients, 429 (86%) could be discharged due to an unremarkable CT examination on-site. A total of 69 patients (14%) had to be transported to a nearby emergency room, with 17 patients (3%) being moved because of challenging clinical management without abnormal CT findings. Transportation to the ED was carried out by an advanced life support Level 1 (ALS1) emergency ground ambulance. On average, the distance to the emergency room amounted to 3 miles. A total of 7 nearby emergency rooms were accessed from the ambulance station. The TBI patients were referred exclusively to one of the three neurosurgical centers in the city of Munich.

### 2.2. Model Structure

**Figure 1:**
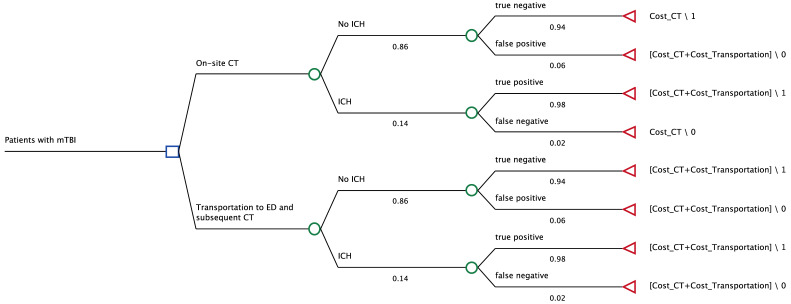
Decision model for our analysis. Either patients were directly examined via CT scanning on-site and, depending on the results, were subsequently discharged or transported to a nearby hospital or they were directly transported to a nearby ED and received a CT scan thereafter. Percentages of ICH in each group were derived from real patient data of the Munich Oktoberfest in the years 2022 and 2023. The sensitivity and specificity of CT for ICH were included in the analysis. ICH, intracranial hemorrhage.

To compare the cost effectiveness of on-site CT scans to the primary transportation and treatment in an ED of patients with suspected head trauma, we constructed a decision model using the decision-making software TreeAge Pro 2024 (Williamstown, MA, USA) (see Figure 1). Two scenarios were considered. First, we used the collected data of patients receiving a CT scan on the event site who, depending on the results, were discharged or transferred to an ED via ambulance transportation for further management and/or follow-up. Second, a hypothetical scenario was implemented in which all patients from the first scenario with CT indication were transported directly from the event to an ED, where the CT scan and further procedures were performed. The accuracy of CT scans for intracranial hemorrhage (ICH) was implemented into the analysis using sensitivity and specificity for detection of ICH, as described below in the section for input parameters. This measure was chosen to also include unnecessary transport to the emergency department, where an ICH was falsely detected by the radiologist on-site. Conversely, false negative examinations were also to be included where patients were discharged from the Sanitätswache despite ICH and were not transported to an emergency department. Overall, the aim was to simulate clinical reality as accurately as possible.

### 2.3. Input Parameters

Sensitivity and specificity for the detection of intracranial hemorrhage (ICH) in computed tomography were derived from the literature [11].

Billing information for CT scanning, as well as ambulance transportation, was collected from German national reimbursement rates [12,13].

To make our approach more perceivable, we calculated costs for international patients at the Munich Oktoberfest based on common billing practice for German private health insurance. Furthermore, we calculated a scenario from the perspective of a major trauma center in the U.S. (University of Rochester, Rochester, NY, USA) using Medicare data [14] and information from the Ambulance Fee Schedule [15] (Urban Base Rate) (see Table 1). All costs were calculated in 2024 the national currencies (i.e., EUR and USD).

### 2.4. Outcomes

On-site CT examination and the direct transport of patients with mTBIs to a nearby ED were compared according to total reimbursements per patient and their differences for each of the three perspectives mentioned above.

### 2.5. Sensitivity Analysis

Deterministic one-way sensitivity analysis (DSA) was performed to identify variables that significantly influence the model outcomes. The DSA was performed from the hypothetical U.S. perspective, as costs for transport distance could easily be implemented, as well as for the German and international perspectives. The ranges for deterministic sensitivity analysis were determined by the 95% confidence interval of the initial probabilities and by ±20% for costs. The range for transport distance was set to 0 to 50 miles. Based on the literature of ICH in mTBI patients presenting to EDs worldwide, the range of abnormal CT findings was determined to be from 3.9% to 33.9% [19].

### 2.6. Imaging

The following scanners were used on-site for mobile CT scanning of the head, maxillofacial region, and/or cervical spine: SOMATOM go.Top (Siemens Healthineers, Erlangen, Germany) in 2022 and SOMATOM Scope (Siemens Healthineers, Erlangen, Germany) in 2023. Only patients who had a cranial CT scan were included in our evaluation; costs for maxillofacial CT and imaging of the cervical spine have not yet been reflected in the billing.

## 3. Results

Average costs per patient for on-site CT accumulated to EUR 243 from the German perspective, EUR 438 for international patients, and USD 160 from the U.S. perspective. Costs for primary transportation to a nearby ED and further diagnostic work-up, including CT, led to average costs per patient of EUR 908 from the German perspective and EUR 1635 for international patients, as well as USD 403 from the U.S. perspective. Therefore, costs from the international perspective were the highest, followed by costs from the German and U.S. perspectives.

For each scenario, on-site CT was associated with lower costs compared to primary transport to a nearby ED, resulting in cost savings of EUR 665 in the German analysis, EUR 1197 for international patients, and USD 242 from the U.S. perspective (see Table 2).

When including only ambulatory transportation for patients with remarkable CT findings, costs only slightly differed without significant changes (see Table 3).

### Sensitivity Analysis Results

**Figure 2:**
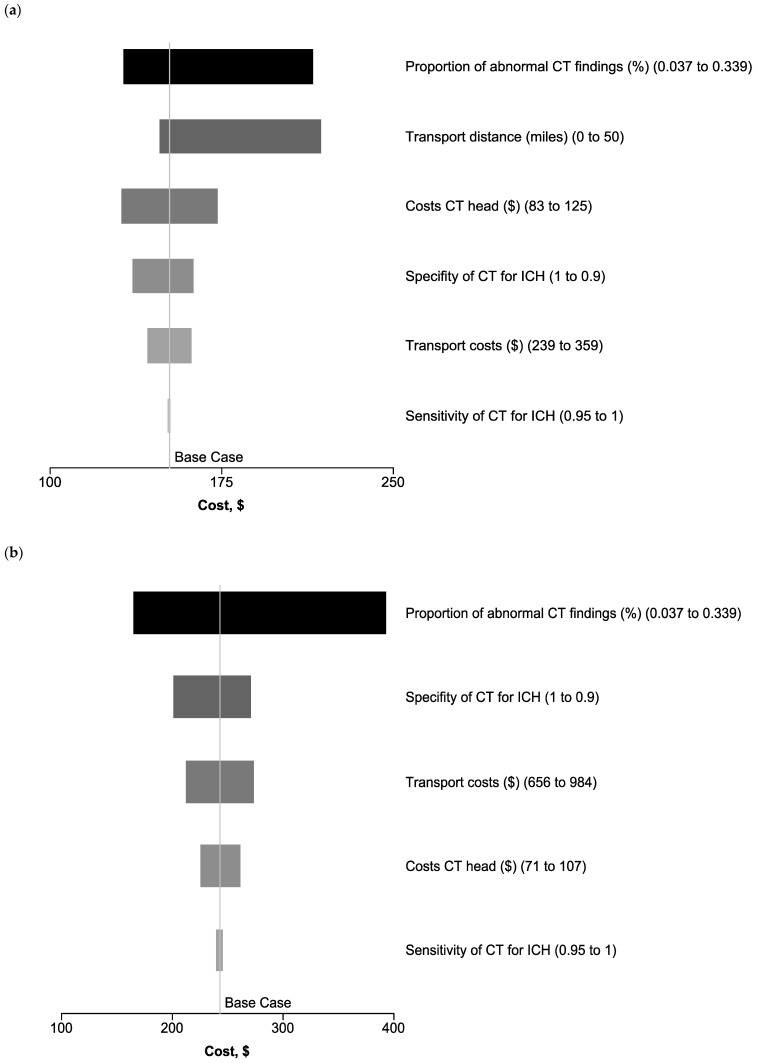

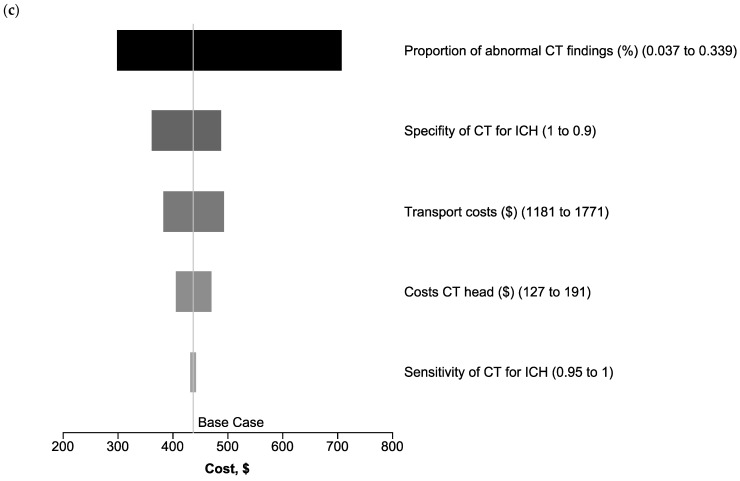
Deterministic sensitivity analyses of on-site CT from a U.S. (**a**) and German (**b**), as well as an international perspective (**c**). Compared to the calculated costs per patient, the proportion of ICH in the patient group, as well as long transport distances, had the strongest impact on average costs per patient from the U.S. perspective. From the German and international perspectives, the proportion of ICH in the patient group remained the strongest impact on the costs, followed by the sensitivity of CT scans for ICH, as well as costs for transportation. ICH, intracranial hemorrhage.

DSA for on-site CT revealed the proportion of patients with ICH as well as long transport distances as having the strongest impact on the average cost per patient. However, even for higher proportions of patients with ICH, the costs for on-site CT examination per patient remained below the average costs for the direct transport of patients to a nearby ED (USD 215 vs. USD 403). Costs for the CT scan itself and the specificity of CT for ICH, as well as the base costs for ambulatory transportation per Current Procedural Terminology codes (CPT codes), only had a minor effect on the costs, followed by the sensitivity of CT scans for ICH, as well as costs for transportation. Again, costs for on-site CT stayed below the average costs for the direct transport of patients to a nearby ED (see Figure 2).

## 4. Discussion

Mass gatherings, as defined by the WHO, are spontaneous or organized events with a large number of attendees that challenge local resources. They have the potential to strain medical resources by affecting the ability of communities to respond to a significant emergency. Therefore, comprehensive planning and resource management are needed to cope with these challenges [20].

Mass gatherings can be divided depending on the occasion, ranging from sports events to concerts to Folkfests, like the Oktoberfest. Each of these presents its own risks, the latter with alcohol use or recreational drugs.

Clinical assessment in intoxicated patients with mTBIs is unreliable, requiring head CT scans, mostly at an ED after emergency transport, thus, potentially delaying the diagnosis or treatment of other life-threatening disorders.

Installation of an on-site CT scanner at a large-scale event, such as the Munich Oktoberfest, can significantly decrease the amount of necessary ambulance transport, reduce overcrowding in EDs, and help triage patients [7].

Previous studies showed that providing medical care directly on-site in the setting of large-scale events, such as music festivals or sports events accompanied by associated events with many visitors, is rewarding by reducing the usage of ambulance transport and the impact on emergency management and health systems [21,22,23,24]. The usage of X-Ray imaging and point-of-care ultrasound at music festivals has been reported [25,26,27]. To our knowledge, no study has yet been conducted analyzing the cost effectiveness of radiologic on-site imaging in this setting.

Our data analysis demonstrated that using a mobile CT scanner directly on-site at a large-scale event like the Munich Oktoberfest is cost effective compared to transporting patients to a trauma center for head CT scanning in different healthcare settings. In all three scenarios, healthcare reimbursements for on-site CT scans were lower than for the direct transport of patients to a nearby ED. The proportion of patients with ICHs had a strong impact on the costs of CT scans on-site. However, based on the literature, a wide range of proportions of ICH was chosen for sensitivity analyses. Costs for the transport of patients to a nearby ED, the specificity of CT for ICH, as well as long distances for the transport of patients to an ED in the U.S. setting had a moderate impact in the sensitivity analyses. However, costs in all sensitivity analyses stayed below the costs for the primary transport of patients to an ED, supporting the results of our base case analyses. Therefore, not only from a medical point of view but also from an economic perspective, it can be recommended to examine patients with mTBIs on-site rather than transporting them to a nearby trauma center for head CT scans.

For our analysis, we used rather conservative reimbursement rates from a U.S. healthcare perspective. For the European perspective, we used German national reimbursement rates, showing that a significant economic value can be achieved by using mobile CT examinations of the head on-site in patients with mTBI/intoxication in the context of the Munich Oktoberfest.

A thorough estimation of the number of expected patients to be examined with CT on-site is recommended for the calculation of the cost effectiveness of such a procedure. However, as our deterministic sensitivity analysis showed, costs for on-site CT examination were still considerably lower than those of the direct transport of patients with suspected ICH to an ED, even for a high proportion of patients with ICH. For the world’s first deployment of a mobile CT scanner at a festival such as the Oktoberfest, we leveraged robust data from the German emergency systems, including the Interdisciplinary Medical Care Capacity Management System (IVENA), alongside extensive experience from emergency departments and rescue services in Munich and Bavaria. This allowed us to utilize what we consider a highly valuable tool with exceptional effectiveness in providing medical support at this large-scale event.

CT operation times were synchronized with the periods of the days and nights in which the most mTBIs are to be expected on-site and the most transport, due to mTBIs, to surrounding EDs were to be expected at Munich’s Oktoberfest.

### Limitations

In order to enable the transferability of our data onto different international healthcare systems, we intentionally did not include additional costs, such as personnel costs, renting costs for mobile CT scanners, setup costs, or costs of electricity, since these factors are highly dependent on the respective countries, where healthcare is provided. Instead, we used reimbursement rates to reflect the actual costs resulting from the healthcare system. This has to be taken into account when calculating the absolute economic benefits of providing on-site CT imaging at large-scale events.

## 5. Conclusions

Our analysis showed that the use of an on-site CT scanner at a large-scale event for patients with mTBIs can be economically beneficial compared to transporting patients directly to an emergency department for head CT scans from German and U.S. healthcare perspectives.

## Figures and Tables

**Table 1:** Input parameters.

**Study Population**		
Patients (total)	498	
Patients being transported to the ED	69 (14%)	
-with abnormal CT findings	52 (11%)	
-because of challenging clinical management	17 (3%)	
**CT scan**		
Sensitivity ICH	0.98 [11]	
Specificity ICH	0.94 [11]	
Reimbursement rate for CT scanning (Germany)	88.31 € [16]	https://www.kbv.de/html/ebm.php, accessed on 9 December 2024.
Reimbursement rate for CT scanning international patients (Germany)	159 €	
CT scan head (CPT Code 70450)	$104 [17]	
**Transportation**		
Reimbursement rate for ALS 1 emergency ambulatory transport (Germany)	820 € [18]	
Reimbursement rate for ALS 1 emergency ambulatory transport of international patients (Germany)	1476 €	
Average distance to the ED (miles)	3	
Ground distance per statute mile (CPT Code A0425)	$8.76 [15]	
ALS1 emergency, advanced life support, ambulance emergency transport (CPT Code A0427)	$272.44 [15]	

Input parameters for our analysis with associated sources. Costs refer to current national billing information. ALS1, advanced life support Level 1; CPT, Current Procedural Terminology.

**Table 2:** Results (all transport).

Scenario	Costs of On-Site CT	Costs of Primary Transport to ER	Difference
Germany	EUR 243	EUR 908	EUR 665
International patient	EUR 438	EUR 1635	EUR 1197
U.S. perspective (Rochester)	USD 160	USD 403	USD 242

Results are displayed as cost per patient. Costs are shown in national currencies.

**Table 3:** Results (only CT-indicated transport).

Scenario	Costs of On-Site CT	Costs of Primary Transport to ER	Difference
Germany	EUR 220	EUR 908	USD 688
International Patient	EUR 385	EUR 1564	EUR 1179
U.S. perspective (Rochester)	USD 152	USD 403	USD 251

Results are displayed as average cost per patient. Costs are shown in national currencies.

## Data Availability

The raw data supporting the conclusions of this article will be made available by the authors upon request, as long as their disclosure is not protected by German law.
